# Identification of the dwarf gene *GmDW1* in soybean (*Glycine max* L.) by combining mapping-by-sequencing and linkage analysis

**DOI:** 10.1007/s00122-017-3044-8

**Published:** 2018-03-17

**Authors:** Zhong-feng Li, Yong Guo, Lin Ou, Huilong Hong, Jun Wang, Zhang-xiong Liu, Bingfu Guo, Lijuan Zhang, Lijuan Qiu

**Affiliations:** 1 National Key Facility for Gene Resources and Genetic Improvement/Key Laboratory of Crop Germplasm Utilization, Ministry of Agriculture, Institute of Crop Sciences, Chinese Academy of Agricultural Science, No. 12 Zhongguancun South Street, Haidian District, Beijing, 100081 People’s Republic of China; 2 College of Agriculture, Yangzi University, Jingzhou, 434025 People’s Republic of China

## Abstract

**Key message:**

*GmDW1* encodes an *ent*-kaurene synthase (KS) acting at the early step of the biosynthesis pathway for gibberellins (GAs) and regulates the development of plant height in soybean.

**Abstract:**

Plant height is an important component of plant architecture, and significantly affects crop breeding practices and yield. Here, we report the characterization of an EMS-induced dwarf mutant (*dw*) of the soybean cultivar Zhongpin 661 (ZDD23893). The *dw* mutant displayed reduced plant height and shortened internodes, both of which were mainly attributed to the longitudinally decreased cell length. The bioactive GA_1_ (gibberellin A_1_) and GA_4_ (gibberellin A_4_) were not detectable in the stem of *dw*, and the dwarf phenotype could be rescued by treatment with exogenous GA_3_. Genetic analysis showed that the dwarf trait of *dw* was controlled by a recessive nuclear gene. By combining linkage analysis and mapping-by-sequencing, we mapped the *GmDW1* gene to an approximately 460-kb region on chromosome (Chr.) 8, containing 36 annotated genes in the reference Willliams 82 genome. Of these genes, we identified two nonsynonymous single nucleotide polymorphisms (SNPs) that are present in the encoding regions of *Gmdw1* and *Glyma.08G165100* in *dw*, respectively. However, only the SNP mutation (T>A) at nucleotide 1224 in *Gmdw1* cosegregated with the dwarf phenotype. *GmDW1* encodes an *ent*-kaurene synthase, and was expressed in various tissues including root, stem, and leaf. Further phenotypic analysis of the allelic variations in soybean accessions strongly indicated that *GmDW1* is responsible for the dwarf phenotype in *dw*. Our results provide important information for improving our understanding of the genetics of soybean plant height and crop breeding.

**Electronic supplementary material:**

The online version of this article (10.1007/s00122-017-3044-8) contains supplementary material, which is available to authorized users.

## Introduction

Ideal plant architecture has recently become a significant breeding objective in many crops (Reinhardt and Kuhlemeier 2002). Height is one of several important components of plant ideotypes, and a relatively shorter stem length contributes to attaining higher yield in crop production (Cooper et al. 1995, 2003). Dwarfism is a desirable characteristic in crop breeding because it confers enhanced resistance to lodging damage from wind and rain and is associated with stable, increased yields by improving the harvest index. For instance, the introduction of semi-dwarf varieties in wheat and rice led to substantial increases in grain yields throughout Asia in the 1960s and 1970s, and prevented many people across the world from starving; this time period is known as the Green Revolution (Peng et al. 1999; Khush 2001; Hedden 2003). In soybean, many semi-dwarf cultivars such as Hobbit87, Charleston, and Apex were also developed. These semi-dwarf cultivars were high yielding and had potential for lodging resistance (Cooper et al. 1995, 2003).

Gibberellins (GAs) regulate diverse biological processes in plant growth and development such as seed germination, stem elongation, leaf expansion, and flowering (Sun and Gubler 2004). Previous studies on dwarf mutants in the model plant species *Arabidopsis thaliana* (Helliwell et al. 1998; Magome et al. 2004) and in rice (Hong et al. 2003; Ji et al. 2014) have revealed that GAs are one of the most important phytohormones determining plant height. Both gibberellin-deficient and -insensitive mutants showed alterations in plant height. For example, a mutation in the *sd1* allele, encoding a gibberellin 20-oxidase gene (GA20oxs), reduced endogenous GA levels and led to the short stature of rice variety IR8 (Sasaki et al. 2002; Spielmeyer et al. 2002). The *sd1* seedlings respond to exogenous GAs, which restore height to that of wild-type plants. A similar case happened to another rice semi-dwarf cultivar, Tan-Ginbozu (*d35*^Tan-Ginbozu^) with a weak allele of the *ent*-kaurene oxidase, contributing to the increase in rice yield in Japan in the 1950s (Itoh et al. 2004). The wheat gene *Rht* (Peng et al. 1999), the maize gene *dwarf*-*8* (*d8*) (Fujioka et al. 1988), and their orthologue *GAI* in *Arabidopsis* (Peng et al. 1997), all encode the DELLA protein, a key component of the molecular GA-GID1-DELLA mechanism controlling plant response to GA (Ueguchi-Tanaka et al. 2005, 2007a, b; Harberd et al. 2009). These mutant alleles always reduce plant height and show reduced responses to GAs. However, the different manner of mutation on the DELLA protein (e.g., SLR1, a rice DELLA protein) can lead to opposite GA response phenotypes: a constitutive GA response slender plant (Ikeda et al. 2001), and a GA-insensitive dwarf (Asano et al. 2009; Hirano et al. 2010).

Mutants play an important role in identifying gene functions in flowering plants. Using spontaneous or artificially induced dwarf mutants, many genes have been cloned and functionally characterized in rice, and many of these are involved in metabolic pathways of plant hormones such as GA, BR (brassinosteroid), and/or strigolactone (SLs) (Itoh et al. 2004; Hirano et al. 2010; Hong et al. 2003; Tong et al. 2009; Lin et al. 2009; Zhou et al. 2013). Within these genes, *D35* encodes the gibberellin biosynthesis enzyme, *ent*-kaurene oxidase, and all shortened internodes of *d35* plants were restored to the wild-type phenotype by the GA_3_ treatment (Itoh et al. 2004). However, for another semi-dominant dwarf mutant, *Slr1*-*d4*, a mutation in the C-terminal GRAS domain of SLR1 caused its reduced responsiveness to GA_3_ (Hirano et al. 2010). BR is another important hormone involved in plant height development. The *d2* mutants, deficient in the downstream biosynthesis pathway of BR, can respond to exogenous BR treatment, and *D2* encoded a cytochrome P450 protein with high similarity to the identified BR synthesis enzymes (Hong et al. 2003). The dwarf and low tillering phenotype of *dlt* was less sensitive to treatment with BRs, since DLT, a new member of the plant-specific GRAS family, positively regulates the BR signaling pathway in rice (Tong et al. 2009). SLs may also take part in rice stem elongation. D27, a novel iron-containing protein, is involved in biosynthesis of SLs, and the reduced plant height phenotypes of *d27* could be rescued to the wildtype by applied strigolactone (Lin et al. 2009). However, *d53* is a rice SL-insensitive mutant and the reduced height does not respond to exogenous SL treatment due to a gain-of-function mutation in the D53 protein, acting as a repressor of SL signaling (Zhou et al. 2013). In soybean, increasing numbers of mutants causing dwarfing have been also identified; these mutants occur following ionizing (gamma rays and fast neutrons) radiation (Zhang et al. 2014; Hwang et al. 2015; Cheng et al. 2016) and/or chemical mutagenesis-generated populations (Li et al. 2017). However, except for the peroxidase-encoding gene underlying the *del3*-*15* locus on chromosome 15 (Hwang et al. 2015), few dwarf genes have been cloned and characterized to date.

In this study, we performed a thorough phenotypic characterization of the *dw* mutant, and reported the molecular identification of the *GmDW1* gene by an integrated approach that involved linkage analysis, mapping-by-sequencing, and allelic variation test, which encodes the key enzyme *ent*-kaurene synthase (KS) that functions in the GA biosynthetic pathway in soybean. Our results indicated that *GmDW1* plays a key role in GA-regulated cell elongation in soybean stem internodes.

## Materials and methods

### Plant materials

The seeds of soybean (*Glycine max*) cultivars Zhongpin 661(Zp661, ZDD23893), Jidou 12 (JD12, ZDD23040), and Zhonghuang 13 (Zh13, ZDD23876), together with eight other soybean accessions for the allelic variations tests in *GmDW1* (Table 8), were obtained from the National Crop Gene Bank, Chinese Academy of Agricultural Sciences. The *dw* dwarf mutant was isolated from an EMS-mutagenized M3 line in the genetic background of Zp661, which was derived from a cross between the soybean cultivar Williams (PI 548631) and Buffalo (PI 424131) (Li et al. 2017).

### Plant hormone treatment and endogenous GA determination

Zp661 and the *dw* mutant were grown in a growth chamber at 25 °C under conditions of 16-h daylight, 8-h darkness, and 75% humidity. Approximately 14 days after emergence (DAE), 1 g (fresh weight) internode tissue from the mutant or wild-type seedlings was harvested, weighed, immediately frozen in liquid nitrogen, and then stored at − 80 °C. The phytohormone extraction and quantitative profiling of GAs (GA_1_, GA_3_, GA_4_, GA_5_, GA_6_, GA_7_, GA_8_, GA_9_, GA_12_, GA_13_, GA_15_, GA_19_, GA_20_, GA_23_, GA_24_, GA_29_, GA_34_, GA_44_, GA_51_, and GA_53_) were performed as described by Chen et al. (2012). These analyses were conducted by the Key Laboratory of Analytical Chemistry for Biology and Medicine of Wuhan University in China.

To assess the response of *dw* to phytohormones, a range of concentrations of GA_3_ from 0 to 1.0 mg/L were applied three times for a week to treat the seedlings with fully open true leaves. Uniconazole (Uni) (a GA biosynthesis inhibitor) treatment was carried out at the same time (Itoh et al. 2004). Soybean seeds with no physical damage were soaked in Uni solution (0.6 mg Uni fully diluted in 1 L of double distilled water) for 2 h, and then transferred to vermiculite for normal germination in a growth chamber. Soybean growth condition was set as mentioned above. For each treatment three repeats were prepared, and 1 week later the effect of hormone on stem expansion was evaluated by measuring seedling length.

### Scanning electron microscopy

To measure cell size, an internode of 14-DAE *dw* and Zp661 seedlings was split in half, fixed in 2.5% glutaraldehyde solution (pH 7.4) for 48 h at room temperature, and then processed according to the manual supplied with the scanning electron microscope (Hitachi, S-3000N).

### DNA extraction, primer design, and sequencing PCR products

A single young leaf was collected from each plant at the V2 stage (one fully expanded trifoliolate). Genomic DNA was extracted using the modified CTAB method (Saghaimaroof et al. 1984), and diluted to a concentration of 20 ng/μL in ddH_2_O. Primers were designed online using Primer3 (http://primer3.ut.ee/) based on the Williams 82 reference genome. PCR reactions (25 µL) were composed of 4 μL genomic DNA (20 ng/μL), 2.5 μL PCR buffer (10×), 2.0 μL dNTPs (2 mmol), 2.0 μL MgSO_4_ (25 mmol), 2.6 μL forward and reverse primers (2 μmol), 0.4 U Kod-Plus-Neo DNA polymerase (TOYOBO, Japan), and sterile water. PCR amplification started with a denaturing step at 94 °C for 3 min, followed by 36 cycles of denaturation at 98 °C for 20 s, annealing at 58–60 °C for 20 s, extension at 68 °C for 50 s, and a final extension at 68 °C for 6–8 min before cooling to 10 °C. PCR products were separated on 2% agarose gels stained with ethidium bromide, visualized in a UV light box, and then sequenced using the Sanger method (Sanger et al. 1977).

### Segregation population and genetic mapping

The F_1_ from a cross between a *dw* plant and a wild-type (Zp661, JD12, or Zh13) plant was self-crossed to generate an F_2_ population for genetic analysis and mapping of the *dw* mutant. The parental lines, ten random F_2_ recessive individuals, and ten wild-type plants from the F_2_ population of *dw* × JD12 were used for bulked segregant analysis. To screen for polymorphic markers, the two parents were genotyped with 567 SSR markers across 20 soybean chromosomes (Song et al. 2010). Together with some newly developed SNP markers, the polymorphic SSR markers were selected for genotyping the two tail bulks and the F_2_ mutant plants derived from a cross of *dw* and JD12. The SSR assay was performed using polyacrylamide gel electrophoresis, as described by Wu and Tanksley (1993). Detailed information on the linkage markers for genetic mapping of the *dw* mutant is shown in Table 1.

**Table 1:** Basic information on the SSR markers and developed SNP markers linked to the *GmDW1* gene on chromosome 8

Primer ID	Forward strand sequence (5′–3′)	Reverse strand sequence (5′–3′)	Physical position (Mb)	Product size (bp)
GMENOD2B^a^ (08-0556)	TAGGCAAAAGACTAAAAGAGTA	GCATGTCATTTTGATTGA	10.19	169
BARCSOYSSR_08_0687^a^ (08-0687)	TCTCACCACCACCCTCTTTC	CCTGCAGCAAAACGTCACTA	12.38	226
BARCSOYSSR_08_0692^a^ (08-0692)	TCTGTTAGCAATTCTTATGTAACCG	TCAATTCTTGTTCACAAATCAATAAA	12.57	171
BARCSOYSSR_08_0706^a^ (08-0706)	GGCTAATTTAAGAAAATTTAAAACACG	AATGTTGATAATAAAATCACATGCTTA	12.89	287
BARCSOYSSR_08_0716^a^ (08-0716)	GGGACAATGTGCGAGGTTAG	AAATTGTTGAACCTTTTATTTTTCA	13.07	279
BARCSOYSSR_08_0762^a^ (08-0762)	CACAAGCAATCCCTGACAGA	CAGAAACCGTGGAAACCCTA	13.95	264
BARCSOYSSR_08_0777^a^ (08-0777)	TCGGCCAATGAGTATACGTG	CACGATGGACTTCACGACAT	14.17	258
Sat_129^a^ (08-0818)	GGGGACTCCCTCTCCAGAAGTAAT	GGGAGCAATTGATAAGTGTGAAAATAAT	14.73	239
BARCSOYSSR_08_0935^a^ (08-0935)	TGGATCGATTGTTTTCCAAGA	AAAAATTATCATGGCAGCCG	16.85	231
BARCSOYSSR_08_0941^a^ (08-0941)	AAGGAACAAGTAAAGGAATCATCA	CACCGCACCTTATATTATTACGAA	16.91	285
SNP08-1^b^	TGCACCAAAACCAGCTCAAT	AGGATCAGAAGGCTTGGGAC	12.61	876
SNP08-2^b^	CCCGGTGCCAATTTTGAAGT	GATCAAACTTGCTCGTGACCA	12.69	833
SNP08-3^b^	TCCTCTCGTCAAAAGCTCCA	CCAAGTGTACAGAGCAATCCTTT	12.85	925
SNP08-4^b^	TGAAAGCCTTGACATTGCGG	GGCAAAAGGAACCCAAGGAT	12.90	703
SNP08-5^b^	TCTAAAGAGCCTACCGTGGG	AAGCAATGCCCCTCAATGTG	13.01	774
SNP08-6^b^	CTGGTGTCAAATTCCCCTGC	AAA GGC ACC GAA CAT CTT GC	13.08	848

^a^The unified or classical name for SSR markers associated with the *GmDW1* locus are displayed

^b^SNP markers were developed based on the SNP mutations indentified in the *dw* genome, as shown in Table 6

### Whole genome resequencing, SNP detection and identification of the candidate interval

A similar strategy to the MutMap^+^ method (Fekih et al. 2013) was used to isolate the *GmDW1* gene. One plant that contained a heterozygous *DW* locus was selected from an M3 line derived from the EMS-mutagenized population of cv. Zp661. This plant was self-crossed to generate an isogenic M4 segregating population. DNA from 45 mutant or 45 wild-type plants was extracted and equally pooled. According to the manufacturer’s instructions (Illumina Inc.), > 5-μg genomic DNA for each pool was prepared for constructing a sequencing library. Paired-end sequencing libraries with an insert size of approximately 500 bp were sequenced on an Illumina HiSeq 2500 sequencer. Variation calling and annotation was conducted following the protocol of Zhou et al. (2015). Theoretically, for the causal SNP allele, the genotype should be mutated in the raw reads from the mutant pool, while partial reads or no reads containing variant target SNP loci should be present in the wild-type pool. The ED (Euclidean distance) method was used to evaluate differences in allele frequencies between the two phenotype pools for each of the identified SNPs along the 20 chromosomes of soybean. Based on the analysis of the ED values of SNPs, several putative linked regions for the *dw* dwarf phenotype were detected (Hill et al. 2013; Su et al. 2016).

### RNA extraction, reverse transcription PCR, and quantitative real-time PCR

Soybean growth condition was set as mentioned earlier in plant hormone treatment and endogenous GA determination. Fresh tissues from 2-week-old (14 DAE) seedlings were collected, immediately frozen in liquid nitrogen, and stored at − 80 °C for RNA extraction. Total RNA from leaves, stem, and root was extracted using an RNA Prep Pure Plant kit (Tiangen Co., Beijing, China), and treated with DNaseI (Thermo Fisher Scientific Inc., Grand Island, NY). cDNA was synthesized using a SuperScript II kit (TaKaRa). Real-time PCR was performed using a SYBR Premix Ex Taq™ kit (TaKaRa) on an ABI 7300 Real-Time PCR System. Three replicates were run for each sample. The soybean *Actin11* gene (*Glyma.18G290800*) was used as the internal control (Cook et al. 2012). Transcript abundance for some GA mechanism-related genes in soybean was measured using primers listed in Table 2. The relative expression level against the *Actin11* gene was quantified using the $2^{{ - \Delta \Delta C_{\text{T}} }}$ method (Livak and Schmittgen 2001).

**Table 2:** GA mechanism-related genes in soybean and the primers for qPCR analysis

Some identified GA mechanism-related genes in plants	Soybean homologs	Primer ID	Forward strand sequence (5′–3′)	Reverse strand sequence (5′–3′)
GAs biosynthesis-related genes from *Arabidopsis* and *Medicago truncatula*				
Copalyl pyrophosphate synthase (CPS) (*AT4G02780*)	*Glyma.19G157000*	CPS-2	ACTGCCACCTTCCCTCTTTC	TGTTTGTCGTTAGTCTCGGAC
GA-20 oxidase (*AT4G25420*)	*Glyma.09G149200*	GA-1	GATAGAGAGACCCTGTGCCT	TGAGAAGCAGAGCAAAACAGAG
	*Glyma.20G153400*	GA-2	TGGCTGCAACGGAAAAGTAA	TAGCCCCATAGCCCTACTCA
*Ent*-kaurene synthase (KS) (*MTR_2G064295*)	*Glyma.08G163900*	RT08-5	ATGTGCTGGCTTTGCGTATT	CCTTGCACTCTCTGGGAACT
GA-responsive genes isolated in *Arabidopsis* or *Medicago truncatula*				
*GID1a* (*MTR_8G035520*)	*Glyma.20G230600*	GR-2	AGTTCCTGTATCCCTGTGCC	TGGCAGGGAAAGAGAAGAGG
*RGA* (*AT2G01570*)	*Glyma.05G140400*	GR-6	CTGGCTCCAAACCATGCTTT	CCCCGGAATAGCCTTGAGAT
	*Glyma.11G216500*	GR-8	TCCCCAGATCGTTACCATCG	TCCCAAGGTACAACTCGGAC
Reference gene				
*The soybean Actin11* gene	*Glyma.18G290800*	Actin11	ATCTTGACTGAGCGTGGTTATTCC	GCTGGTCCTGGCTGTCTCC

## Results

### Characterization of *dw* mutants

**Fig. 1:**
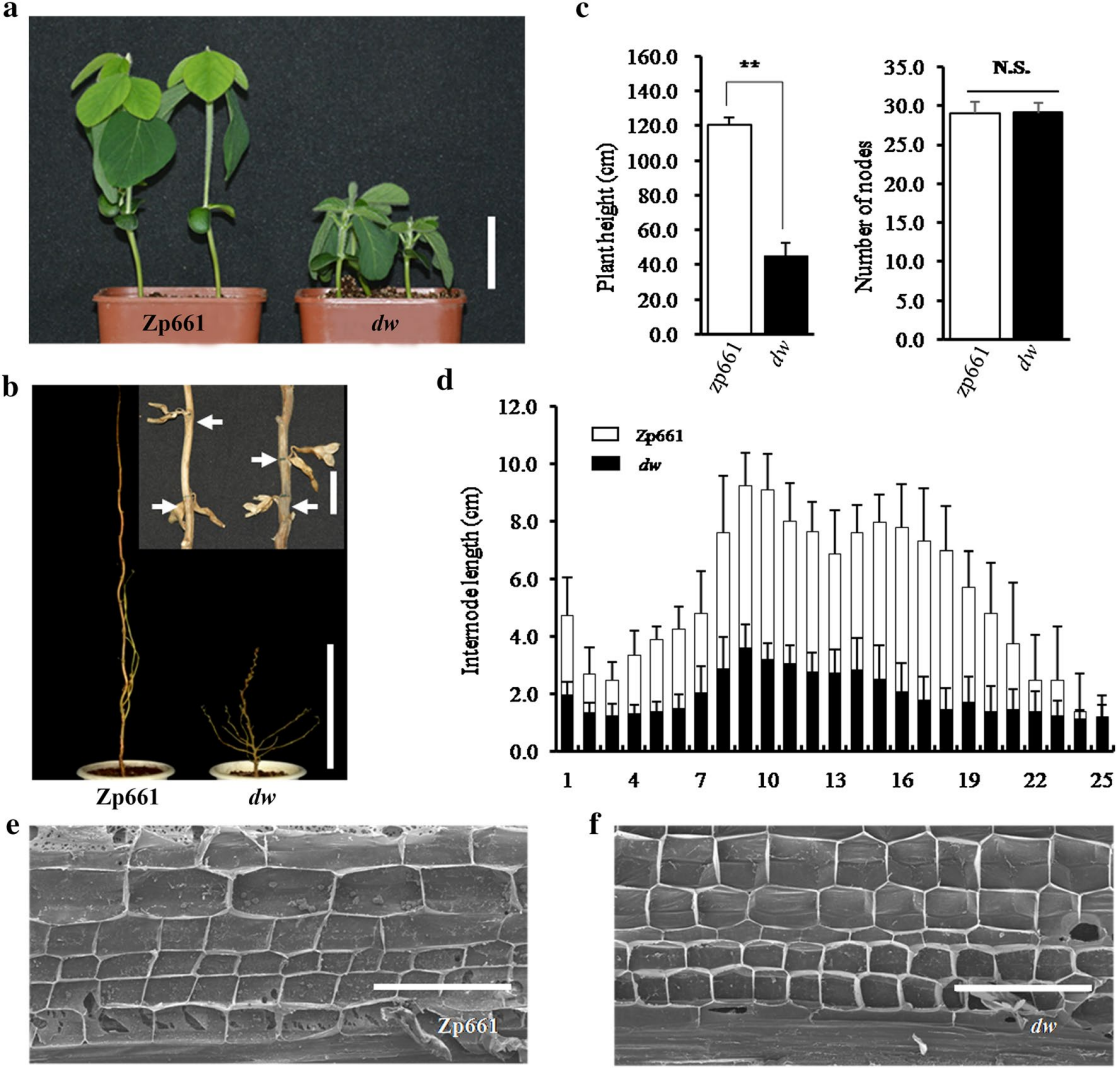
Phenotypic characterization of the soybean *dw* mutant. **a** The plant height of the *dw* mutant and the parent Zp661 at the seedling stage (2 weeks after emergence). **b** Phenotype of wild type and *dw* at maturity. White arrows indicated the nodes bearing soybean pods. **c** The plant height and the number of nodes on the stem of the mutant and the parent at maturity. **d** Comparison of all internode lengths for *dw* and wild-type plants at maturity (*n* = 15 plants). Longitudinal sections of the first internodes on the stem of the mutant (**f**) and the parent (**e**) at the V2 stage (one fully expanded trifoliolate). Scale bar is 5 cm in **a**, 4 cm for local area magnification and 45 cm for overview in **b**, 200 µm in **e**–**f**, respectively. A Student’s *t* test indicated a significant difference (*n* = 15 plants) in **c**. **P < 0.01; *NS* not significant. All data are given as mean ± SD

The *dw* mutant arose from an EMS-mutagenized M2 population as described by Li et al. (2017). From emergence to maturity stages, the mutant displayed a significant, constant decrease in the length of the main stem equivalent to 40% of the size of the mature wild-type Zp661 plants; the mutants also had dark green leaves (Fig. 1a, b). Compared with wild type, the *dw* plant was not significantly different in total node number on the main stem, but had a relatively consistent reduction in the internode length of 60% (Fig. 1c, d). The longitudinal sections of middle internodes at the seedling stage were observed with a microscope. In *dw* plants, cell width was similar to wild type, however, the cell length was much shorter than wild type (Fig. 1e, f). Therefore, both reduced plant height and shortened internodes in *dw* are mainly attributed to the longitudinally decreased cell length instead of a decrease in the number of cells.

### The dwarf mutant is deficient in the GA biosynthesis pathway

**Fig. 2:**
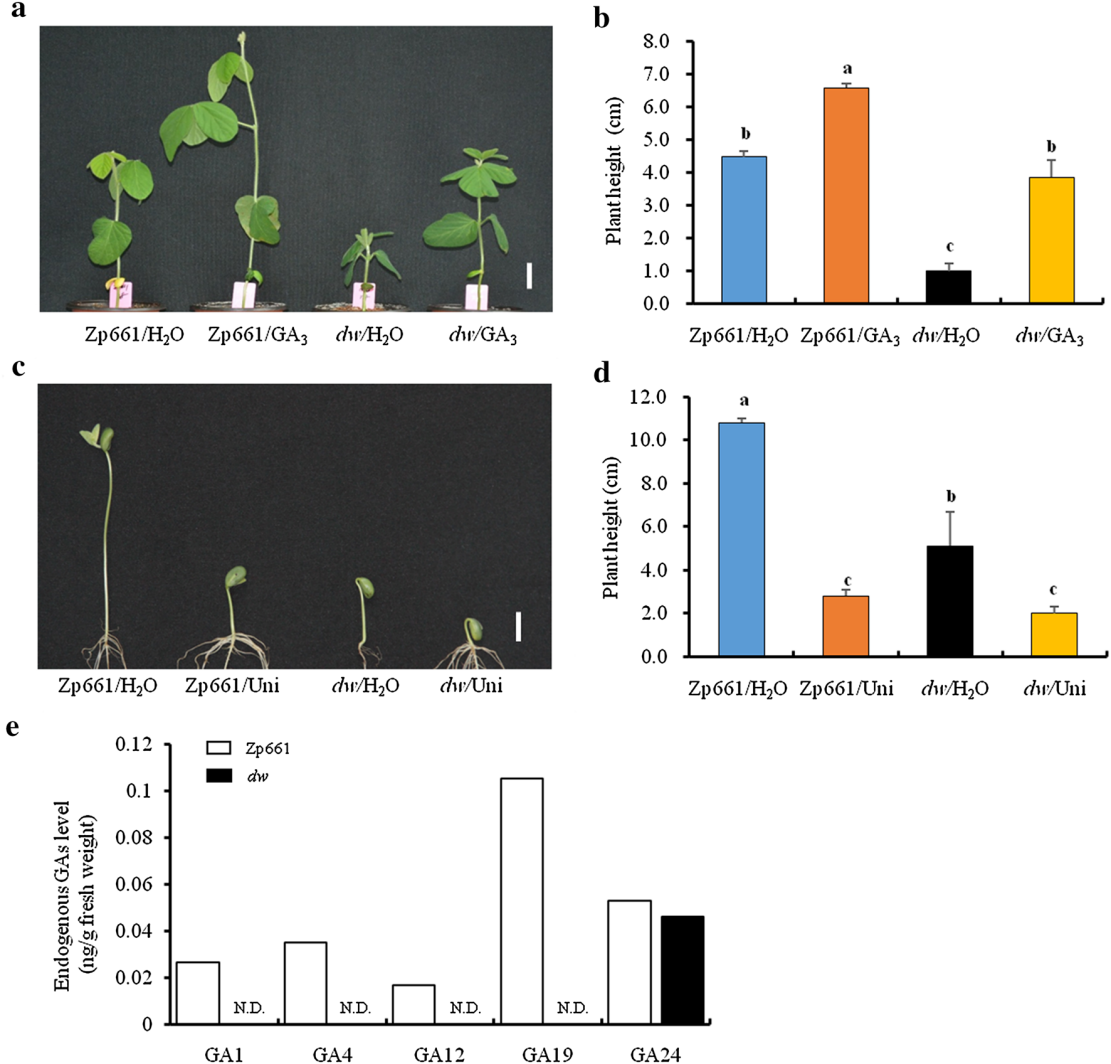
*dw* is a GA-deficient soybean mutant. The morphological phenotypes (**a**) and the statistical data of plant height (**b**) of wild-type and *dw* plants 1 week past the exogenous GA_3_ (0.1 mg/L) application. Phenotypes (**c**) and the statistical data of plant height (**d**) of 1-week-old elongated *dw* and Zp661 seedlings after treatment with 0.6 mg/L Uni (uniconazole, a GA_3_ biosynthesis inhibitor). **e** Determination of endogenous GA levels in the first internodes of 14-day-old *dw* and Zp661 plants. The water treatment was used as the control and the scale bar is 2 cm for **a** and **c**. *ND* not detectable. Data for **b** and **d** are based on a growth chamber experiment using a randomized complete block design with three replications. The statistical significance of the phenotypic differences among different treatments was evaluated using one-way ANOVA. Bars superscripted by different small letters are significantly different at the 5% probability level

Various factors result in stunted stem growth (Hirano et al. 2010; Tong et al. 2009; Zhou et al. 2013). To determine possible reasons for the *dw* dwarf phenotype, a series of hormone treatments were performed. GA_3_ at concentrations of 0–1 mg/L promoted longitudinal stem internode extension in the *dw* plant, and restored the dwarf mutant to the wild-type phenotype (Fig. 2a, b, Supplemental Fig. S1), whereas BR and IAA showed no effect on stem elongation of *dw* (data not shown). Uni (Uniconazole), a GA biosynthesis inhibitor, was also used to treat the *dw* and Zp661 seedlings. In contrast to GA_3_, Uni treatment resulted in a greater reduction in the shoot length of wild-type seedlings compared to mutants (Fig. 2c, d). Endogenous GA levels in the stem internodes from both wild-type and *dw* plants were determined using gas chromatography–mass spectrometry (GC–MS). Bioactive GA_1_ and GA_4_, as well as their immediate precursors GA_12_, GA_19_, and GA_24_, were detected in wild-type plants, but only GA_24_ was detected in the *dw* mutant, suggesting that the *dw* phenotype was associated with substantially decreased levels of bioactive GA (Fig. 2e). Taken together, these results confirmed that *dw* has a lower active gibberellin level in the stem, and that it is a GA biosynthesis-deficient mutant.

### Genetic analysis of the *dw* mutant

Plant height in crop plants, such as wheat, maize, and soybean, is mostly determined by a set of quantitative trait loci (QTLs) (Singh et al. 2016; Teng et al. 2013; Zhang et al. 2004). To determine whether the extreme dwarf phenotype was controlled by a single gene or locus, the *dw* mutant was crossed with three varieties that had normal phenotypes, including the wild-type parent Zp661 and the cultivars JD12 and Zh13. All F_1_ plants displayed normal phenotypes resembling the wild-type parent. In the three F_2_ populations, mutant individuals were easy to distinguish because of the extreme dwarf stature and dark green leaves. Out of an F_2_ segregated population from *dw* × Zp661, plant height of 97 and 31 F_2_ plants were similar to the wild-type parent and mutant-type, respectively, corresponding to the expected 3:1 segregation ratio for a single recessive gene (*χ*^2^ = 0.04, *P* = 0.84) (Table 3). Similarly, a 3:1 ratio of wild-type individuals to mutant individuals was also detected in the F_2_ population derived from a cross between the *dw* mutant and JD12 (*χ*^2^ = 2.09, *P* = 0.15)/Zh13 (*χ*^2^ = 0.28, *P* = 0.59) (Table 3). These data indicated that the *dw* mutation behaved in a monogenic recessive manner.

**Table 3:** Genetic analysis of the *dw* dwarf phenotype in F_2_ segregated populations from three crosses

Cross	Phenotype of F_1_ plants	Wild type (plants)	Dwarf^a^ (plants)	Total (plants)	$\chi^{2}_{3:1}$	*P* ^b^
*dw* × Zp661	Wild type	97	31	128	0.04	0.84
*dw* × JD12	Wild type	231	92	323	2.09	0.15
*dw* × Zh13	Wild type	146	53	199	0.28	0.59

^a^Dwarf plants were identified by visual inspection based on a phenotype of reduced plant height, shortened internodes, and dark green leaves

^b^*P* > 0.05 is considered significant

### Mapping of the *GmDW1* gene by whole genome resequencing

Using next-generation sequencing (NGS), several strategies have been developed, and used to rapidly identify the causal mutations responsible for important traits induced by chemical mutagenesis (Deschamps et al. 2012). Here, an M3 plant carrying a heterozygous *GmDW1* locus was selfed to form an isogenic M4 segregated population consisting of 53 dwarf and 161 wild-type individuals. The DNA pool was generated by bulking 45 mutants or 45 wild-type individuals, and was subsequently subjected to high-throughput whole genome resequencing (Illumina HiSeq 2500 platform), which yielded 454 million and 415 million 2 × 126 bp read pairs for the mutant- and wild-type pools, respectively. Over 94% of the total reads were properly and uniquely mapped to the Williams 82 reference genome, corresponding to average nucleic genome coverage of > 50-fold (Table 4). Based on alignment to the Williams 82 draft genome sequence, 283,626 SNPs were identified in the mutant pool, and 294,871 SNPs were present in the wild-type pool (Table 4). Between the two resequenced samples, a total of 47,535 high quality SNPs were detected for further analysis, indicating that a large number of SNP variations are present in the mutated *dw* genome.

**Table 4:** Basic data for two DNA pools by whole genome resequencing

Samples	No. of total clean reads	Mapped (%)^a^	Average depth (*x*)	Genomic coverage (%)	No. of SNPs^b^
Wild type	415,681,226	95.89	50	99.39	294,871
Mutant type	454,898,978	94.01	53	99.15	283,626

^a^Number of clean reads mapped to the Williams 82 reference genome divided by the total number of clean reads × 100

^b^The number of base changes between the resequenced wild-type or mutant DNA pool and the Williams 82 reference genome

The Euclidean distance (ED) algorithm has been proven to be useful in obtaining the genetic distance to the associated genes or QTLs (Hill et al. 2013; Su et al. 2016). To map the *GmDW1* gene controlling the dwarf phenotype of the *dw* mutant, we used the ED method to compute allele frequency differences for each SNP locus along the physical map of soybean between the two DNA pools (Hill et al. 2013; Su et al. 2016). ED value analysis, with a threshold of 0.259, revealed that a total of six intervals with a physical distance of 93 kb–5.7 Mb, were possibly linked to the dwarf trait in *dw*. Out of these candidate-mapping regions, Chr. 7 and Chr. 8 each had one interval (designated *locus7*-*1* and *locus8*-*1*), and Chr. 14 and Chr. 15 each had two intervals (designated *locus14*-*1, locus14*-*2, locus15*-*1, and locus15*-*2*) (Table 5). However, just part of the identified SNPs in *locus7*-*1*, *locus8*-*1*, and *locus14*-*1* result in nonsynonymous substitution of amino acids in the deduced protein sequence, suggesting that these three regions are associated with the dwarf phenotype in *dw* plants.

**Table 5:** Mapping regions associated with the dwarf phenotype of *dw* mutants identified by whole genome resequencing of two bulked DNA pools

The names of the linked regions	Chromosome ID	Physical position of candidate intervals			Locations of the identified SNPs in the corresponding mapping regions		
		Interval start (bp)	Interval stop (bp)	Interval length (kb)	Intergenic region^a^	Gene^a^ (nonsynoymous)^b^	Up- or downstream^a^
*locus7*-*1*	Chr. 07	513,840	606,834	92	0	2 (1)	0
*locus8*-*1*	Chr. 08	8,716,986	14,491,037	5774	12	68 (16)	51
*locus14*-*1*	Chr. 14	8,075,271	9,355,387	1280	14	2 (1)	8
*locus14*-*2*	Chr. 14	24,821,398	27,119,121	2297	5	0	1
*locus15*-*1*	Chr. 15	21,601,429	22,103,288	501	4	0	0
*locus15*-*2*	Chr. 15	35,344,325	36,214,614	870	6	1 (0)	1

^a^The number of identified SNPs located in the open reading frame, intergenic region, and up- or downstream. The number of SNPs resulting in nonsynonymous substitution of amino acids in the deduced protein sequence is identified by ^b^

### Validation of the candidate interval for *GmDW1* by linkage mapping

**Fig. 3:**
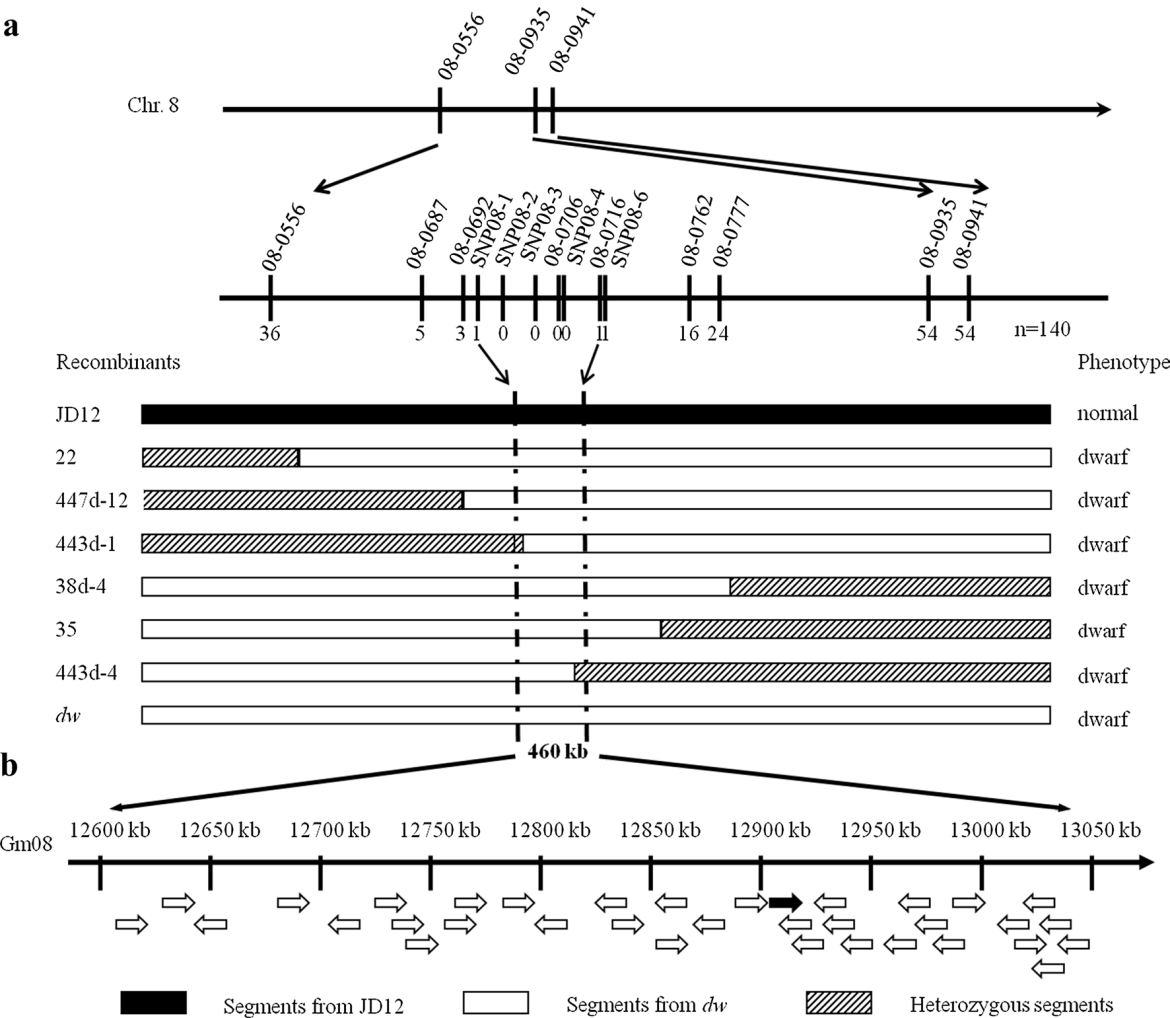
Genetic and physical mapping of *GmDW1*. **a** Genetic mapping of the *GmDW1* locus. Using some key recombinants screened from the F_2_ segregated population originating from *dw* × JD12, the location of the *GmDW1* locus was narrowed down to a 460-kb region bounded by marker SNP08-1 and SSR marker 08-0716 on chromosome 8. The numerals below the markers indicate the number of identified recombinants. **b** Relative physical position of the *GmDW1* locus. Thirty-six annotated ORFs (open reading frame) existed in the 460-kb fine-mapping interval according to the Williams 82 reference genome. Black arrow indicated the position of *GmDW1* in **b**. 08-0716, BARCSOYSSR_08_0716; 08-0556, GMENOD2B; 08-0935, BARCSOYSSR_08_0935; 08-0941, BARCSOYSSR_08_0941; 08-0687, BARCSOYSSR_08_0687; 08-0692, BARCSOYSSR_08_0692; 08-0706, BARCSOYSSR_08_0706; 08-0762, BARCSOYSSR_08_0762; 08-0777, BARCSOYSSR_08_0777

To further screen for the causal interval from those candidate regions, linkage analysis was also conducted based on the F_2_ population of *dw* × JD12. A total of 567 SSR markers (Song et al. 2010) evenly distributed on 20 chromosomes were used to screen the *dw* mutant, JD12, a wild-type pool HP (high plants), and a dwarf pool DP (dwarf plants). Bulked segregant analysis allowed for linkage of the *dw* locus to four SSR markers on Chr. 8: 08-0935 (BARCSOYSSR_08_0935), 08-0941 (BARCSOYSSR_08_0941), 08-0556 (GMENOD2B), and 08-0818 (Sat_129) (Fig. 3a). Thirty-seven dwarf F_2_ plants were individually genotyped using these linkage markers, and 3 or 12 recombinants were identified between SSR marker 08-0556 and/or 08-0818, or 08-0941 and the *Gmdw1* gene, respectively. Thus, the dwarf gene was initially mapped within an interval of 6.7 Mb between the marker 08-0556 (GMENOD2B) and 08-0941 (BARCSOYSSR_08_0941). Interestingly, our linkage analysis showed good correspondence with the locus on Chr. 8 (*locus8*-*1*) resulting from resequencing the two bulked DNA pools, and confined the *Gmdw1* gene to a 4.3-Mb physical interval.

### Fine mapping of the *GmDW1* gene

To further narrow down the candidate-mapping region, additional linkage markers on Chr. 8 were screened, and used to analyze a total of 140 F_2_ individuals with the dwarf phenotype from the F_2_ population of *dw* × JD12. Polymorphic markers 08-0692 (BARCSOYSSR_08_0692), 08-0706 (BARCSOYSSR_08_0706), and 08-0716 (BARCSOYSSR_08_0716) were applied to genotype the identified 90 recombinants between 08-0556 and 08-0941 from the 140 F_2_ dwarf individuals, and 3, 0, and 1 recombination events were detected, respectively, indicating that the candidate gene was mapped to the segment with a physical distance of approximately 500 kb between marker 08-0692 and 08-0716 (Fig. 3a). By analyzing the resequenced mutant genome, we found that there were only nine SNPs in the mapping region for *dw* (Table 6). Based on these SNP mutations, six SNP markers (SNP08-1-SNP08-6) were developed and used to genotype those four recombinant plants. Only one recombinant was detected between SNP08-1 or SNP08-6 and the *GmDW1* gene, respectively, while the markers SNP08-2, SNP08-3, and SNP08-4 cosegregated with the dwarf phenotype in the F_2_ population, which was confirmed based on 50 F_2_ mutant plants from a cross of *dw* × Zh13. Accordingly, we were finally able to restrict the location of this gene to a 460-kb interval between markers SNP08-1 and 08-0716 (BARCSOYSSR_08_0716) on Chr. 8 (Fig. 3a). According to the Williams 82 reference genome, the fine-mapping region contains 36 predicted genes (Fig. 3b), which are listed in Table 7.

**Table 6:** Nine identified SNPs in the 500-kb mapping region containing the *GmDW1* allele in the *dw* genome and marker development

The ID of developed markers based on the corresponding SNP locus	SNP			The wild-type pool		The mutant pool		SNP effect and target gene
	Physical position in Gm08 (bp)	Reference^a^	Variation^b^	Genotype^c^	Reads depth^d^	Genotype^c^	Reads depth^d^	
	12,598,498	G	A	G, A	24, 15	G, G	28, 0	Intergenic
SNP08-1	12,613,790	C	T	C, T	18, 5	T, T	0, 26	Nonsynonymous, *Glyma.08G162100*
SNP08-2	12,686,354	G	T	G, G	26, 0	G, T	9, 6	Upstream
SNP08-3	12,847,120	T	A	T, A	50, 6	A, A	0, 63	Upstream
SNP08-4	12,903,104	T	A	T, T	43, 0	A, A	0, 41	Nonsynonymous, *Glyma.08G163900*
	12,969,978	G	A	G, A	10, 3	G, G	13, 0	Downstream
SNP08-5	13,012,790	C	A	C, C	37, 3	C, A	26, 5	Nonsynonymous, *Glyma.08G165100*
	13,055,558	G	A	G, A	32, 18	G, G	24, 0	Upstream
SNP08-6	13,077,287	C	T	C, C	34, 0	T, T	0, 37	Nonsynonymous, *Glyma.08G165800*

^a^The genotypes of the SNP locus in the Williams 82 reference genome, while ^b^ represents the genotypes of the corresponding mutated locus present in the mutant- or wild-type pool genome. ^c^ Homozygous or heterozygous genotype (e.g., “G,G” vs “G,A”) of the SNP locus was identified in the wild-type pool and mutant-type pool genome

^d^The number of clean reads covering each of genotypes at the SNP locus

**Table 7:** Thirty-six predicted genes in the 460-kb fine-mapping interval of *GmDW1* in Gm08 according to the Williams 82 reference genome

Gene ID	Functional annotation in the Phytozome database
*Glyma.08G162200*	Methylated RNA-binding protein 1
*Glyma.08G162300*	Nucleoprotein TPR-related
*Glyma.08G162400*	Aspartic protease CDR1-related
*Glyma.08G162500*	DNA-3-methyladenine glycosylase I/DNA-3-methyladenine glycosidase
*Glyma.08G162600*	39S ribosomal protein L15, mitochondrial
*Glyma.08G162700*	Peroxidase/Lactoperoxidase
*Glyma.08G162800*	Zinc finger CCCH domain-containing protein 5
*Glyma.08G162900*	Metacaspase-5-like
*Glyma.08G163000*	E3 ubiquitin-protein ligase RGLG2-like
*Glyma.08G163100*	NAC domain-containing protein 20-related
*Glyma.08G163200*	MYB-like DNA-binding protein
*Glyma.08G163300*	Uncharacterized protein
*Glyma.08G163400*	Uncharacterized protein
*Glyma.08G163500*	MYB family transcription factor APL-like
*Glyma.08G163600*	Uncharacterized protein
*Glyma.08G163700*	Succinate-semialdehyde dehydrogenase, mitochondrial-like
*Glyma.08G163800*	Cell cycle control protein 50
*Glyma.08G163900*	*Ent*-kaurene synthase, chloroplastic-like
*Glyma.08G164000*	50S ribosomal protein L7/L12-like, mitochondrial
*Glyma.08G164100*	IMP dehydrogenase/Inosinic acid dehydrogenase
*Glyma.08G164200*	Carbohydrate-binding X8 domain-containing protein
*Glyma.08G164300*	Uncharacterized protein
*Glyma.08G164400*	Zinc transporter 1-like
*Glyma.08G164500*	G-protein coupled receptor
*Glyma.08G164600*	Bifunctional L-3-cyanoalanine synthase/cysteine synthase D1-related
*Glyma.08G164700*	Metal tolerance protein 10-like
*Glyma.08G164800*	Metal tolerance protein 10-like
*Glyma.08G164900*	Cullin binding (Cullin_binding)/UBA-like domain (UBA_4)
*Glyma.08G165000*	Defense-like protein 1-related
*Glyma.08G165100*	Transglycosylase SLT domain (SLT)
*Glyma.08G165200*	Uncharacterized protein
*Glyma.08G165300*	Uncharacterized protein
*Glyma.08G165400*	Phosphoglycerate kinase, cytosolic-like
*Glyma.08G165500*	Phosphoglycerate kinase 1, chloroplastic-like
*Glyma.08G165600*	DEAD-box ATP-dependent RNA helicase 36-like
*Glyma.08G165700*	Histone-like transcription factor CCAAT-related

### Candidate gene analysis of the *dw* dwarf mutant

**Fig. 4:**
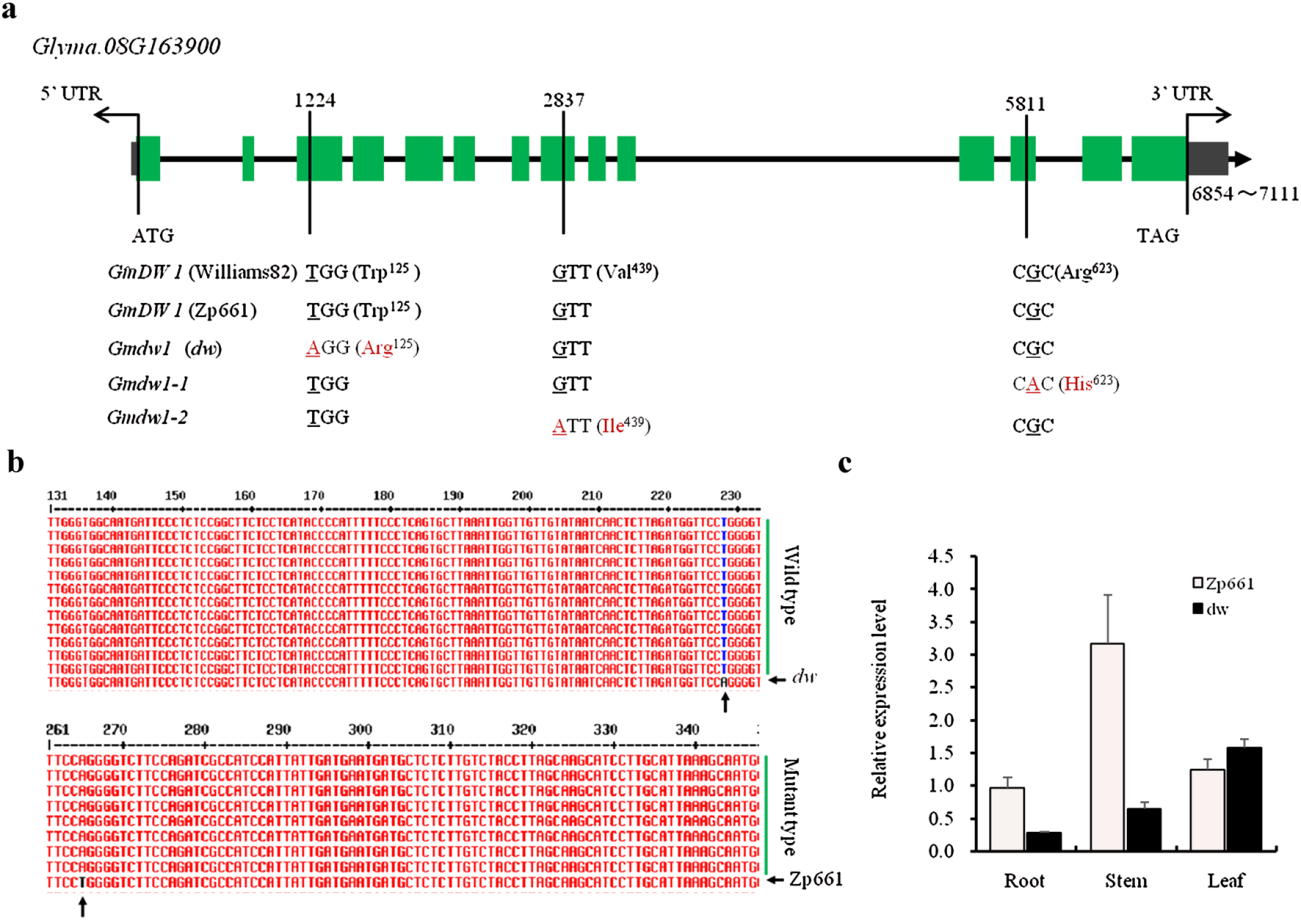
Genetic correlations between the dwarf phenotype in *dw* and *GmDW1* (*Glyma.08G163900*). **a** Genomic sequence of the *GmDW1* allele among *dw*, Zp661, and the Williams 82 reference genome was compared; **a** T-to-A change in the third exon was detected in *dw*, and two additional allelic variations (*Gmdw1*-*1* and *Gmdw1*-*2*) in *GmDW1* were also screened from soybean accessions. **b** The SNP locus (T>A) in *Glyma.08G163900* was linked to the mutant phenotype in *dw*, when genotyping each individual in the resequenced mutant or wild-type pool. **c** Relative expression level of *GmDW1* was detected by qPCR, with data normalized to *Actin11* levels (*n* = 3) (Cook et al. 2012), in different tissues including stem, leaf, and root from 2-week-old *dw* and Zp661 plants

To rapidly isolate the causal mutation responsible for the *dw* mutant, we analyzed the two sequenced DNA samples. For the mutated *dw* genome, however, we only identified seven SNPs in the 460-kb candidate interval (Table 6). Of these SNPs, two were located in exons of *Glyma.08G163900* (named *GmDW1*) and *Glyma.08G165100*, respectively, and formed missense substitutions of the amino acid sequence, while the others were distributed in genes downstream, upstream, or in the intergenic region (Table 6). The indels were also analyzed (data not shown); however, no variations were discovered in the 460-kb fine-mapping interval of the *GmDW1* gene. Therefore, *Glyma.08G165100* and *GmDW1* were the main candidates for the *dw* mutant. According to gene function annotation in the Phytozome database, *Glyma.08G165100* encodes a transglycosylase SLT domain-containing protein, and *GmDW1* encodes KS in soybean. KS is an important enzyme in the upstream biosynthesis pathway of GA, and deficiency in endogenous gibberellin level always results in a dwarf phenotype. In addition, no other GA biosynthesis-related genes were predicted in the candidate-mapping region (Table 7). Further analysis of the clean reads covering the two mutations in the mutant pool revealed 26 wild-type reads out of 31 for the SNP mutation (C>A) in *Glyma.08G165100*. However, 41 reads with the mutation (T>A) in *GmDW1* were detected, which was confirmed when genotyping every individual in the mutant pool (Table 6, Fig. 4b). Taken together with the observed result from GA_3_ treatment, *GmDW1* is the candidate gene for the *dw* mutant.

*GmDW1* contains 14 exons and 13 introns with a 2768-bp transcript encoding 834 amino acids. Analysis of the predicted protein sequence revealed several conserved domains. There is a terpene_cyclase_plant_C1 domain of 522 aa from aa 285–806. A terpene synthase family metal-binding domain is located at aa 501-756. The region of aa 34–815 contains an *ent*-kaurene-16 synthase domain.

In an attempt to identify sequence variations in the candidate gene, genomic sequence corresponding to the ORF and the promoter region of *GmDW1* in the wild type and the *dw* mutant was amplified and sequenced. Besides a T-to-A change in the third exon from BSA (bulked segregant analysis) sequencing, a second SNP mutation (A2416G) and a 3-bp deletion (AAA) were also identified in the 6th intron of *Gmdw1* in the *dw* mutant, but only the single missense point mutation (T1224A) in exon 3 caused an amino acid substitution at residue 125, from Trp to Arg in the *Gmdw1* gene (Fig. 4a).

Tissue expression analysis of the parental line Zp661 at the seedling stage revealed that *GmDW1* was expressed in various tissues including root, stem, and leaf, with the highest level in stem (Fig. 4c).

### Allelic variation in *GmDW1* associated with plant height

To identify the association between plant height and allelic variations in *GmDW1*, we analyzed the resequencing data from 57 wild and cultivated soybean accessions and identified two additional recessive alleles (Li et al. 2013; Lam et al. 2010; Kim et al. 2010). One allele, designated *Gmdw1*-*1*, had a G-to-A change at nucleotide 5811 that resulted in an amino acid substitution at residue 623 from Arg to His in the soybean cultivar ZDD23269 (Li et al. 2013) (Fig. 4a, Table 8). The second, designated *Gmdw1*-*2*, was also a G-to-A change at nucleotide 2837, leading to a substitution of Val with Ile at residue 439 (Fig. 4a). The two alleles were present simultaneously in the wild accession ZYD02878 (designated the *Gmdw* genotype) (Li et al. 2013) (Table 8).

**Table 8:** Phenotypic analysis and allelic variations in *GmDW1* from soybean accessions

Materials^a^	Sequence variation and genotype	SNP effect	Plant height at maturity (cm)^c^	*P* value^d^	Average internode length (cm)^c^	*P* value^d^
Cultivated soybean accessions						
ZDD23269	G-to-A change at nucleotide 5811/*Gmdw1*-*1*	Arg to His at residue 623	102.1 ± 4.3 (*n* = 10)	–	5.2 ± 0.2	–
ZDD23893 (Zp661)	Wildtype/*GmDW1*	Wildtype	130.9 ± 4.7 (*n* = 10)	< 0.0001	5.8 ± 0.4	0.0003
ZDD02315	Wildtype/*GmDW1*	Wildtype	160.0 ± 11.8 (*n* = 5)	0.0111	8.8 ± 1.2	0.0330
ZDD12910	Wildtype/*GmDW1*	Wildtype	171 ± 24.1 (*n* = 5)	0.0101	6.6 ± 0.9	0.0455
ZDD03651	Wildtype/*GmDW1*	Wildtype	180.6 ± 10.4 (*n* = 5)	< 0.0001	8.4 ± 0.2	< 0.0001
Wild soybean accessions						
ZYD02878	G-to-A change at nucleotide 2837 and 5811/*Gmdw*^b^ (*Gmdw1*-*1* and *Gmdw1*-*2*)	Val to Ile at residue 439 and Arg to His at residue 623	7.9 ± 1.7 (*n* = 6)	–	1.4 ± 0.3	–
ZYD03687	Wildtype/*GmDW1*	Wildtype	46.0 ± 15.2 (*n* = 10)	< 0.0001	5.2 ± 1.3	< 0.0001
ZYD04569	Wildtype/*GmDW1*	Wildtype	34.5 ± 9.7 (*n* = 15)	0.0006	3.1 ± 0.8	0.0011
ZYD04638	Wildtype/*GmDW1*	Wildtype	54.2 ± 13.6 (*n* = 6)	0.0004	4.4 ± 1.2	0.0012

^a^Cultivated and wild soybean accessions are given a “ZDDxxxxx” or “ZYDxxxxx” number, respectively, and conserved in the National Crop Gene Bank, Chinese Academy of Agricultural Sciences

^b^The *Gmdw* genotype contained both the *Gmdw1*-*1* and the *Gmdw1*-*2* allele

^c^For each accession, 5–10 plants (*n*) were measured for plant height and internode length. All data are given as mean ± SD

^d^*P* values for differences between the soybean accession with *GmDW1* genotype and the soybean accession with *Gmdw1*-*1* or/and *Gmdw1*-*2 allele* were generated by a Student’s *t* test

To evaluate the plant height for the different *GmDW1* genotypes, we grew the soybean accessions in the field in Hainan province (18.14°N, 109.31°E), or in Beijing (39.97°N, 116.33°E). Cultivars with *GmDW1* and *Gmdw1*-*1* genotypes had plant heights of 130.1–180.6, or 102.1 cm, respectively (Table 8), in the spring of 2012 in Beijing. A similar trend was observed when the wild soybean accessions with *GmDW1* and *Gmdw* (having both the *Gmdw1*-*1* and *Gmdw1*-*2* allele) genotypes were grown in the winter of 2011 in Hainan province: the plant height was 34.5–54.2, or 7.9 cm (Table 8). The *Gmdw1*-*1* and *Gmdw* genotypes showed consistent decreases in plant height phenotypes in both cultivars and wild accessions, despite different genetic backgrounds, suggesting that the sequence variations in the *GmDW1* allele are associated with the dwarf trait in *dw* mutants. Taken together, the results obtained from map-based cloning, genetic, and phenotypic analysis of allelic variation in *GmDW1* in the wild and cultivated soybean accessions strongly indicate that the *GmDW1* gene is responsible for the dwarf phenotype of *dw*.

### Expression analysis of GA metabolic pathway-related genes in soybean

**Fig. 5:**
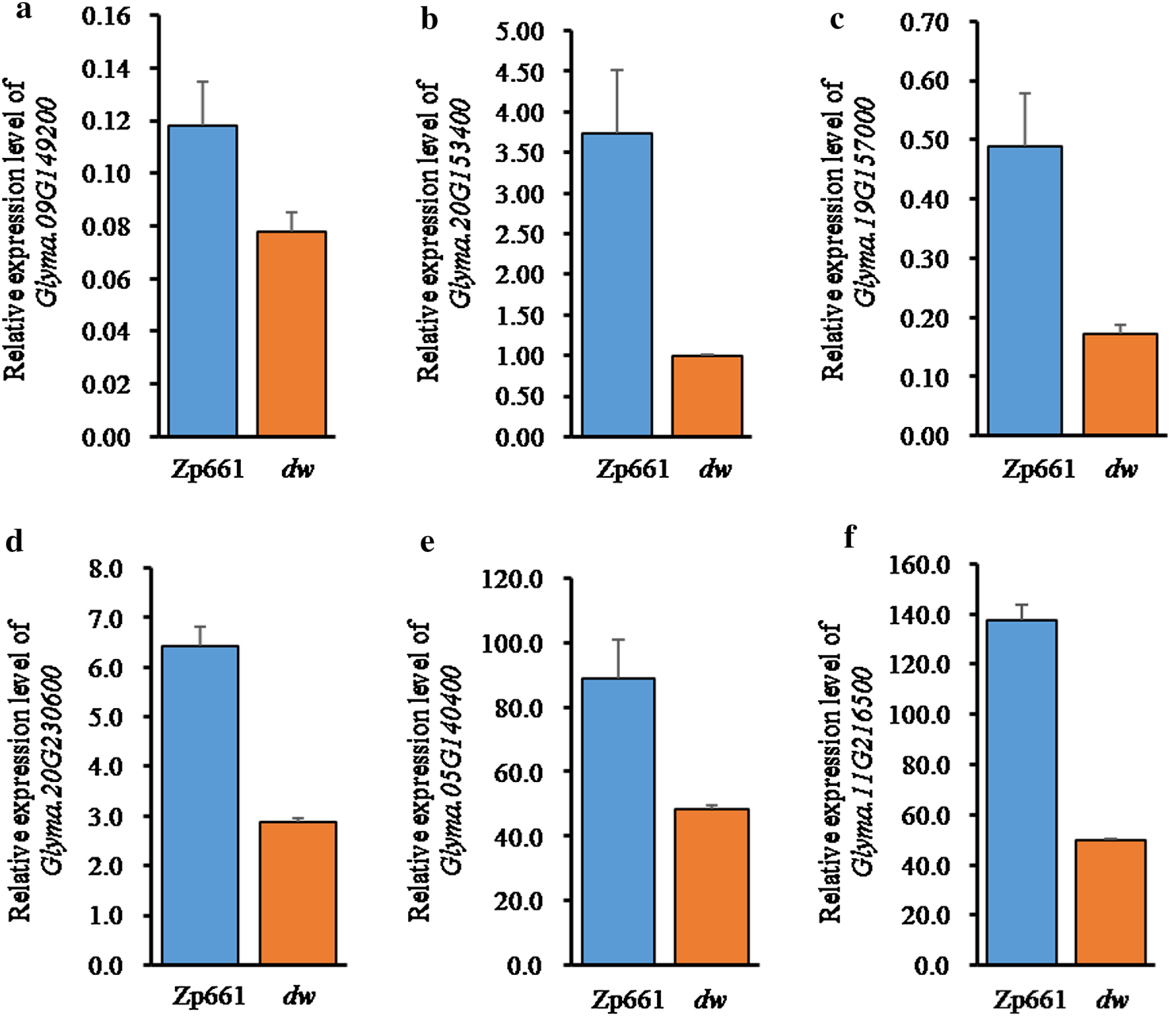
The relative expression of GA metabolic pathway-related genes in *dw* and the parental line Zp661. The expression level of GA biosynthesis-related genes GA-20 oxidase (**a** *Glyma.09G149200*, **b** *Glyma.20G153400*) and CPS (Copalyl pyrophosphate synthase) (**c** *Glyma.19G157000*) homologues in soybean was examined in stems of 14-day-old *dw* and Zp661 seedlings. **d**–**f** The relative expression of GA response-related genes *GID1a* (**d** *Glyma.20G230600*) and *RGA* (**e** *Glyma.05G140400*, **f** *Glyma.11G216500*) homologues in soybean in stems of two-week-old *dw* and Zp661 plants. The soybean *Actin11* gene (*Glyma.18G290800*) was used as the internal control (Cook et al. 2012), and three replicates were performed for each of the genes in **a**–**f**. The relative transcript abundance for these genes in a-f against the soybean *Actin11* gene (*Glyma.18G290800*) was quantified using the $2^{{ - \Delta \Delta C_{\text{T}} }}$ method (Livak and Schmittgen 2001). All the primers for qPCR in **a**–**f** are shown in Table 2

To assess whether relative expression level of GA biosynthesis pathway genes in the *dw* mutant changes, we downloaded the predicted amino acid sequences of *Arabidopsis* CPS (Copalyl pyrophosphate synthase, *AT4G02780*), and GA-20 oxidase (GA20oxs, *AT4G25420*) from the National Center for Biotechnology Information (NCBI). BLAST analysis against the current assembly of the Williams 82 genome was performed using the NCBI Protein BLAST (https://blast.ncbi.nlm.nih.gov/Blast.cgi). The retrieved soybean genomic DNA sequences putatively encoding proteins with high identity to *Arabidopsis* homologs were predicted for GA biosynthesis pathway genes in soybean. As a result, five gene models, including three for CPS, and two for GA20oxs were selected. Of these, three soybean genes (*Glyma.19G157000*, *Glyma.09G149200*, and *Glyma.20G153400*) were expressed in young stem tissues (listed in Table 2). The expression levels of CPS and GA20oxs-encoding genes were lower in stems of *dw* than in the wild-type plant (Fig. 5a–c). Using the same method, we examined the relative expression of some GA response-related genes including *Medicago truncatula GID1a* (*MTR_8G035520*) homolog (*Glyma.20G230600*), and *Arabidopsis RGA* (*AT2G01570*) homologs (*Glyma.05G140400* and *Glyma.11G216500*) in soybean (Table 2). Compared with the wild-type plant these genes also showed substantially decreased expression in stems of *dw* (Fig. 5d–f).

## Discussion

### *dw* is a new GA-deficient soybean mutant

There are many reasons for the dwarf phenotype in plants. In this study, we demonstrated that *dw* is a GA biosynthesis-deficient soybean mutant through exogenous application of GA_3_ and its synthesis inhibitor. The identity of *dw* was further confirmed by determination of endogenous GAs. Meanwhile, compared with the wild type, a relatively reduced inhibition effect of uniconazole on shoot growth was observed in the *dw* seedlings, which is likely related to the way uniconazole impairs GA biosynthesis as a competitive inhibitor of *ent*-kaurene oxidase (Izumi et al. 1985). Besides GAs, other phytohormones, such as BR, SLs, and IAA, may also be involved in regulating plant height (Tong et al. 2009; Lin et al. 2009; Zhou et al. 2013; Woodward and Bartel 2005). However, in the present study, the dwarf phenotype of *dw* was not rescued by BR or IAA treatment. The *dw* mutant is quite different from a few other soybean dwarf mutants that have been intensively studied recently. Of them, the *Gmdwarf1* mutant slightly responded to GA_3_ treatment (Zhang et al. 2014), while dwarfism of *Gmdwf1* could not be rescued by GA_3_ application (Cheng et al. 2016). The FN dwarf mutant, screened from an FN-mutagenized M4 population of Williams 82, began to display abnormal plant height after the V2 stage (Hwang et al. 2015), however, the *dw* mutant in our study showed a consistent dwarf phenotype from the cotyledon expansion stage to maturity. Plant height is generally controlled by node number on the main stem and internode length. We also found that the significantly decreased cell length contributed to the shortened internode, resulting in the sharp decrease in plant height of *dw* compared to wild type, while other dwarf soybean mutants mainly exhibited a substantial change in node number, internode length, or both (Hwang et al. 2015; Cheng et al. 2016). Furthermore, we mapped the *GmDW1* gene to a 460-kb region on Chr. 8, in which no other dwarfing genes have been isolated in soybean.

The GA biosynthesis pathway has been studied extensively in the model organisms *Arabidopsis thaliana* and rice, and the majority of genes encoding key enzymes for each step have been identified (Hedden and Phillips 2000; Olszewski et al. 2002; Sun and Gubler 2004). On the contrary, few GA synthesis pathway-related genes have been isolated in soybean. Here, we determined that an *ent*-kaurene synthase (KS)-encoding gene, functioning at the early step of GA biosynthesis, is responsible for the dwarf phenotype in *dw*, suggesting a conserved function of KS genes in the GA biosynthesis pathway across different plant species.

### Combination of NGS and linkage mapping accelerates the identification of target genes

Forward genetics, for instance, positional cloning is successful for isolating candidate genes of diverse traits, but is labor-intensive and time-consuming (Salvi and Tuberosa 2005). With increasingly high-throughput and decreasing cost, next-generation sequencing (NGS) technology coupled with the growing number of sequenced genomes, has been widely applied to biological research. NGS has been proven to be efficient in identification of candidate genes and SNP discovery in *Arabidopsis* (Ashelford et al. 2011; Leshchiner et al. 2012; Hartwig et al. 2012), rice (Abe et al. 2012), soybean (Zhou et al. 2015), barley (Mascher et al. 2014), and other plant species (Islam et al. 2016). Several mapping strategies based on NGS, such as mapping-by-sequencing or direct resequencing, have been developed, and enable rapid detection of causal mutations responsible for target traits differentiating the mutant from the wild type. However, this method always leads to a few false positive candidate intervals (Ashelford et al. 2011; Abe et al. 2012; Hwang et al. 2015), which also happened in the present study: six putative regions were identified to be linked to the dwarf phenotype of *dw*. Consequently, when NGS was combined with linkage mapping in our work, it was easier to exclude those false loci, and successfully anchor the causal mutation responsible for the dwarf phenotype of *dw* from 36 predicted genes in a short period, greatly reducing the input of labor and time. Our study provides an efficient strategy that has high potential for accelerating the identification of target genes located in a centromeric region in the model plant species such as rice, or in complex non-model genomes such as soybean with a relatively small number of recombinants generated from a segregated population, which will promote the development of functional genomics in crop plant species.

#### **Author contribution statement**

LQ supervised the experiment and revised the manuscript. ZFL, HH, and LO performed the research. ZFL and YG analyzed the data. ZFL wrote the draft manuscript. YG and JW assisted in editing the manuscript. HH, ZXL, BG, and LZ managed the field research and plant propagation. All authors read and approved the final manuscript.

## Electronic supplementary material

Below is the link to the electronic supplementary material.

Supplementary material 1 (DOC 9823 kb)
