# Supplementary material for: Identification of the dwarf gene GmDW1 in soybean (Glycine max L.) by combining mapping-by-sequencing and linkage analysis
Source: Theor Appl Genet. 2018 Mar 17;131(5):1001–16. doi: 10.1007/s00122-017-3044-8 (PMC5895683; doi:10.1007/s00122-017-3044-8)
Supplement: Supplementary file 1 — Supplementary material 1 (DOC 9823 kb) [file 122_2017_3044_MOESM1_ESM.doc]

**Identification of the dwarf gene *GmDW1* in soybean (*Glycine max* L.) by combining mapping-by-sequencing and linkage analysis**

**Theoretical and Applied Genetics**

Zhong-feng Li ^1^, Yong Guo ^1^, Lin Ou ^2^, Huilong Hong^1^, Jun Wang^2^, Zhang-xiong Liu^1^, Bingfu Guo^1^, Lijuan Zhang^1^, Lijuan Qiu ^1^ **^*^**

^1^ National Key Facility for Gene Resources and Genetic Improvement/Key Laboratory of Crop Germplasm Utilization, Ministry of Agriculture, Institute of Crop Sciences, Chinese Academy of Agricultural Science, Beijing 100081, P.R. China; ^2^ College of Agriculture, Yangzi University, Jingzhou 434025, P.R. China.

**^*^** To whom correspondence should be addressed.

**Corresponding author:** Lijuan Qiu

**E-mail:** [qiulijuan@caas.cn](mailto:qiulijuan@caas.cn)


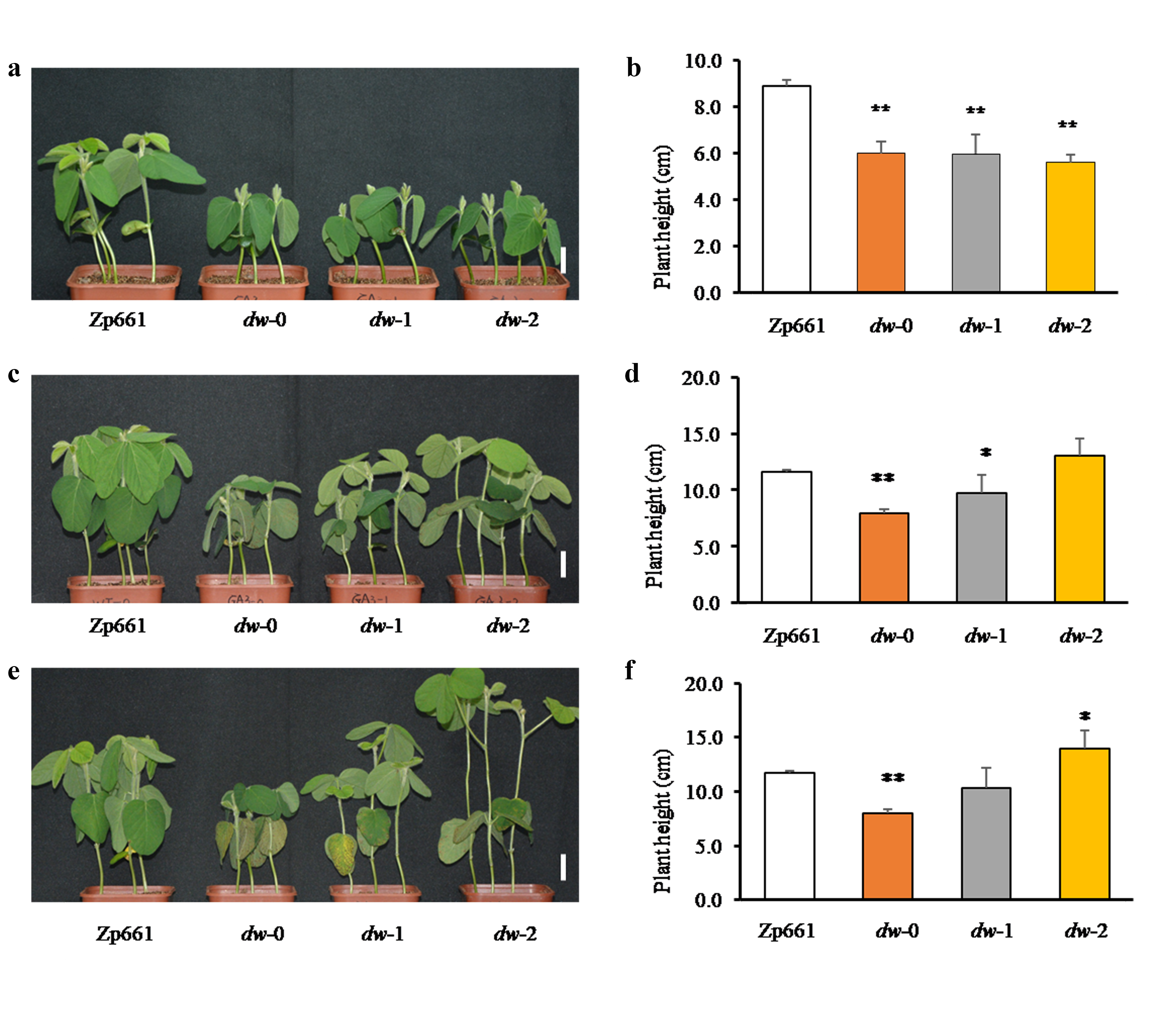


**Supplemental Fig. S1** Application of different concentrations of exogenous GA_3_ to *dw*. Exogenous GA_3_ at different concentrations was applied to *dw*, and plant height was measured after 0 days (a-b), 7 days (c-d), and 10 days (e-f), respectively. *dw*-0, *dw*-1, *dw*-2 indicated 0, 0.1, or 1 mg·L^-1^ GA_3_ application, respectively. Zp661 was sprayed with water in a, c, and e. Scale bar in a, c, and e represents 2 cm. A Student’s *t*-test was used to evaluate the statistical significance of the phenotypic differences between different treatments and the wild-type Zp661. Each sample contained five individuals. ***P* < 0.01; **P* < 0.05
